# Artificial Intelligence in Multiphoton Tomography: Atopic Dermatitis Diagnosis

**DOI:** 10.1038/s41598-020-64937-x

**Published:** 2020-05-14

**Authors:** Pedro Guimarães, Ana Batista, Michael Zieger, Martin Kaatz, Karsten Koenig

**Affiliations:** 1 Saarland University, Chair for Clinical Bioinformatics, Campus E2.1, 66123 Saarbruecken, Germany; 2 Saarland University, Department of Biophotonics and Laser Technology, Campus A5.1, 66123 Saarbruecken, Germany; 3 SRH Wald-Klinikum Gera, Strasse des Friedens 122, 07548 Gera, Germany; 4 JenLab GmbH, Johann-Hittorf-Strasse 8, 12489 Berlin, Germany

**Keywords:** Machine learning, Optical imaging, Diagnosis, Medical imaging, Skin diseases

## Abstract

The diagnostic possibilities of multiphoton tomography (MPT) in dermatology have already been demonstrated. Nevertheless, the analysis of MPT data is still time-consuming and operator dependent. We propose a fully automatic approach based on convolutional neural networks (CNNs) to fully realize the potential of MPT. In total, 3,663 MPT images combining both morphological and metabolic information were acquired from atopic dermatitis (AD) patients and healthy volunteers. These were used to train and tune CNNs to detect the presence of living cells, and if so, to diagnose AD, independently of imaged layer or position. The proposed algorithm correctly diagnosed AD in 97.0 ± 0.2% of all images presenting living cells. The diagnosis was obtained with a sensitivity of 0.966 ± 0.003, specificity of 0.977 ± 0.003 and F-score of 0.964 ± 0.002. Relevance propagation by deep Taylor decomposition was used to enhance the algorithm’s interpretability. Obtained heatmaps show what aspects of the images are important for a given classification. We showed that MPT imaging can be combined with artificial intelligence to successfully diagnose AD. The proposed approach serves as a framework for the automatic diagnosis of skin disorders using MPT.

## Introduction

The human skin is affected by many prevalent diseases, typically widespread across the population and with an immense impact on the patients’ day-to-day life. However, the similarity between lesions hinders disease diagnostics.

Atopic Dermatitis (AD) is the most common chronic inflammatory disease, with prevalence up to 20% in developed countries. It often develops during early childhood, and is recurred throughout the patient’s lifespan^1^, highly influencing their and their families quality of life^2^. AD is characterized by eczematous lesions. However, clinical features manifested are considerably heterogenous, varying with respect to lesion distribution, morphology, intensity, and duration^1,3^. This heterogeneity often hinders the clinician ability to correctly diagnose it.

Multiphoton tomography (MPT) allows non-invasive, label-free, sub-cellular, and 3D-resolved imaging of the human skin. It can simultaneously provide information on tissue morphology and metabolism with high lateral and axial resolutions. Morphology contrast is achieved by the endogenous fluorophores autofluorescence (AF) and collagen second-harmonic generation (SHG). Metabolic information is obtained using nicotinamide adenine dinucleotide and nicotinamide adenine dinucleotide phosphate (NAD(P)H) AF lifetime. During energy production, NAD(P)H undergoes oxidation/reduction mediated by proteins^4,5^. Based on its AF lifetime, free and protein-bound components of NAD(P)H can be separated^5^. The ratio between these components is an indirect measure of the cell’s metabolic activity^4,6^.

The potential of MPT for skin diagnosis is clear and it has already been demonstrated for diseases such as basal and squamous cell carcinomas^7–10^, skin melanoma^10–12^ and even AD^13–17^. Cell irregularities, subcellular changes, like perinuclear accumulation of mitochondria, and metabolic changes are some of the findings observed in the inflamed skin of AD patients^14,15^. Nonetheless, its potential has yet to be fully realized.

In recent years, artificial intelligence-based approaches for image analysis have been exponentially researched and developed. Convolutional neural networks (CNNs) form a subset of deep learning algorithms that take advantage of the relationships between neighboring pixels combining them in consecutive layers to represent exponentially more complex patterns. CNNs are the de-facto networks for image analysis, and have shown strong diagnostic performance when applied to medical images from different fields such as cardiology^18,19^, neurology^20,21^, gastroenterology^22,23^, and dermatology^3,24,25^.

In this study, we demonstrate the feasibility of artificial intelligence for the diagnosis of AD from MPT images. We introduce an accurate and reliable deep learning algorithm for the identification of images with living cells and subsequent diagnosis, removing any operator dependency. We showed that these approaches may be used to fully explore the potential of MPT. This initial work serves as framework for other skin diseases.

## Material and Methods

### Data Source

Data collection was conducted by JenLab GmbH in collaboration with the SRH Wald-Klinikum Gera. This study was approved by the Ethics Committee of the Medical Association of Thuringia, Germany (EUDAMED No: CIV- 17-12-022506) and was conducted following the principles of the World Medical Association Declaration of Helsinki. Informed consent was obtained from all participants.

The analyzed image dataset contains 3,663 patches pulled from a total of 21 image z-stacks acquired from 10 different anonymized subjects. Up to three non-overlapping volumes of the forearm were imaged per subject (two stacks average). In total, six subjects were diagnosed with AD and four were healthy volunteers. A detailed distribution of all the patches according to class is shown in Table 1.

**Table 1:** Detailed distribution of image patches according to class.

	Healthy	Atopic Dermatitis	Total
With Living Cells	566	853	1,419
Without Living Cells	1,018	1,226	2,244
Total	1,584	2,079	3,663

**Figure 1:**
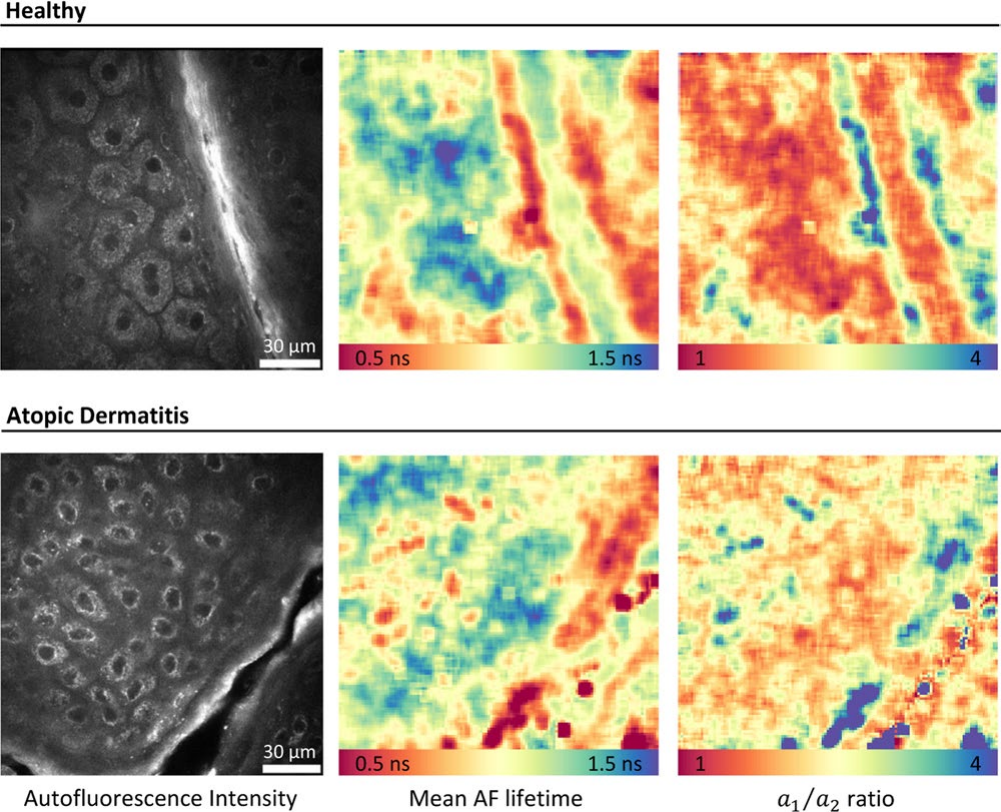
Examples of the three image types to be concatenated for input to the deep learning model. Left – autofluorescence (AF) intensity image; Middle – mean AF lifetime; Right – ratio between the short (${a}_{1}$) and long (${a}_{2}$) relative contributions of AF lifetime components. FLIM images were analyzed using the commercial software SPCImage v8.0 (Becker & Hickl GmbH, Berlin, Germany).

Each z-stack consists of sequential 160 × 160 µm^2^ *enface* images of the skin obtained with 5 µm step in between them, up to a depth of 100 µm. Image resolution was about 300 nm laterally and 1–2 µm axially. After initial fluorescence lifetime analysis, each image was cropped to nine 200 × 200 pixels patches (equivalent to an area of 62.5 × 62.5 µm^2^), with three concatenated channels, consisting of AF intensity, mean AF lifetime, and the ratio between the short (*a*_1_) and long (*a*_2_) relative contributions of AF lifetime components. Examples of the three input channels are shown in Fig. 1. Each patch was also evaluated by an independent expert to detect the presence of living cells. Subjects of both sexes were included, with ages varying between 19 and 78 years old for AD patients and between 27 and 53 for healthy volunteers.

### *In vivo* MPT Imaging Acquisition of the Human Skin

*In vivo* image acquisition was performed using the multiphoton tomograph MPT*flex*-CARS (JenLab GmbH, Berlin, Germany). This device has been described in detail elsewhere^26^. Briefly, this is a movable and flexible MPT imaging device composed by an optoelectronic housing that encompasses the laser and other optoelectronic components, an articulated mirror-arm to guide the laser light, and a flexible 360° scan-detector head. Endogenous fluorophores were excited using an 80 MHz tunable near-infrared (NIR) Ti:Sapphire 100 femtosecond (fs) oscillator, tuned at 800 nm. A high numerical aperture (NA) objective (40×1.3 NA) focused the laser light onto the sample and collected generated signals in reflection geometry. Galvanometric *x-y* scanners and a *z*-stepper motor enable *x-y-z* imaging. A photomultiplier tube (PMT) coupled with a time-correlated single photon counting (TCSPC) module for fluorescence lifetime imaging (FLIM) detected fluorescence. Simultaneously, a second PMT detected second-harmonic generation (SHG) signals from collagen. With this device additional acquisition of coherent anti-stokes Raman scattering (CARS) signals is possible^26^.

### Fluorescence Lifetime Analysis

FLIM images were analyzed using the commercial software SPCImage v8.0 (Becker & Hickl GmbH, Berlin, Germany). AF lifetime data was retrieved by fitting it histogram to a bi-exponential function in the form:

1 $$F(t)={a}_{1}{e}^{\frac{-t}{{\tau }_{1}}}+{a}_{2}{e}^{\frac{-t}{{\tau }_{2}}},$$

where $F(t)$ is the AF intensity at time $t$, and ${a}_{1}$ and ${a}_{2}$ are the relative contribution of the AF lifetime components ${\tau }_{1}$ and ${\tau }_{2}$, respectively. Pixel binning was applied to guarantee the necessary number of photon-counts for accurate fitting. After the selection of the fitting parameters, all FLIM images of the skin were analyzed using batch processing, without user intervention.

AD-induced changes were evaluated depth-wise based on the ratio between $${a}_{1}$$ and $${a}_{2}$$ (*i.e*., $${a}_{1}/{a}_{2})$$ and the tissue mean AF lifetime ($${\tau }_{m}$$), obtained as:

2 $${\tau }_{m}={a}_{1}{\tau }_{1}+{a}_{2}{\tau }_{2},$$

where ${a}_{1}+{a}_{2}=1$. The analysis of the living cell layers was performed after the depth-wise alignment of the s*tratum granulosum* of all z-stacks. The average mean AF lifetime and ${a}_{1}/{a}_{2}$ ratio were computed and recorded at each depth for AD and control subjects. Being composed by dead cells (corneocytes), the s*tratum corneum* was disregarded in this analysis. Statistical differences between healthy and AD-affected skin were assessed using the non-parametric test Mann-Whitney U test (*p* < 0.5).

Pixel resolved images of mean AF lifetime and ${a}_{1}/{a}_{2}$ ratio were exported and concatenated with the AF intensity.

### Atopic Dermatitis Automatic Diagnosis

Although the diagnostic possibilities of MPT imaging have been shown^7–16^, currently the analysis of these images is still largely operator dependent. Factors such as different regions-of-interest and analysis parameters, lead to substantially different results. Here, we propose a fully automatic protocol capable of showing the full potential of these methods. For this reason, deep learning as an end-to-end approach is the perfect fit.

#### Deep learning Models

Image z-stacks are captured from different depths, imaging several layers and covering areas with and without living cells. Previous works have reported changes in the metabolism and morphology of living cells in AD^13,14^. Our approach starts by identifying whether these cells are present in a given image, and if so, evaluates if the imaged skin is diseased. As so, two different CNN models have been developed and trained.

Deep learning models work in contrast with traditional machine-learning approaches: features are not engineered and fed for classification, instead, they are computed incrementally by the model itself. It can create, layer to layer, progressively more complex abstract representations (*i.e*., features), having no theoretical restraint to learn any representation. Nonetheless, this requires a large amount of training data. Deep learning models are prone to overfitting, especially with limited training data, *i.e*., the model learns from patterns that are specific to the training and do not generalize to other data. To overcome this, three actions were taken: (i) we used artificial data augmentation by image rotation, horizontal and vertical image reflection, and scaling, (ii) dropout regularization added to the last fully connected layer of the models (the removal of random nodes approximates the behavior of an ensemble of multiple network architectures that has been shown to reduce overfitting^27^), and (iii) we fine-tuned pre-trained CNNs, *i.e*., transfer-learning. Pre-trained weights are used to initialize the network, thus improving the stability and performance of the model. In this work, we used DenseNet architecture^28^, with weights pre-trained on ImageNet^29^, a dataset with over 14 million labelled images belonging to about 22,000 classes. We adapted the model by replacing the last fully connected and output layer.

#### Model Training and Testing

The performance of the proposed algorithm was evaluated using subject-wise leave-one-out cross-validation. We performed a total of 10 cross-validation runs dividing the data into train, tune and test sets. To ensure complete independency between all the sets, for each run the composition of each set was changed, *i.e*. a new model was created. All images from one of the subjects were left out for testing, the images from another were left out for tuning, while the remainder images were used for training. Each subject was selected only once for testing and could not ever be simultaneously in the testing and training sets. Thus, there is no selection bias by assigning specific testing sets.

Only the training set is artificially augmented. The models were fine-tuned using stochastic gradient descent with a small learning rate and binary cross-entropy was defined as the objective function. Class dependent weighting of the objective function was applied in training to account for class unbalance. The test set is classified using the best performing hyperparameter combination, as assessed by grid-search with early-stopping, evaluated in the tuning set. The evaluated hyperparameters include dropout rate, learning rate, and momentum. All the tested software was implemented in Python v3.5. Models were implemented with Tensorflow v2.0^30^.

#### Variational Dropout for Uncertainty Estimation

For the application of neural networks in the medical field it is imperative to quantify uncertainty, and model output and confidence are not directly correlated^31^. In this study, variational dropout was applied to estimate uncertainty. The idea is to run dropout during testing and perform multiple prediction calls^31,32^. As aforementioned, by turning off several units of the network at random, the multiple calls approximate the behavior of an ensemble of multiple models^27^. The results can then be interpreted as a Bayesian probability distribution^31,32^. In our implementation we performed 50 prediction calls averaging the probabilities obtained. Uncertainty was obtained as the standard deviation of those probabilities. Bayesian probability and variational dropout gives us a mathematically sound approach for uncertainty estimation without forfeiting computational cost or prediction accuracy^31^.

#### Deep Taylor Decomposition

One of the main drawbacks of the application and acceptance of non-linear methods such as deep learning and some other machine-learning approaches in the medical field, is the ‘*black box*’ nature of these methods.

Several approaches exist to improve the interpretability of deep learning methods^33^. In this work, deep Taylor decomposition was applied^34^. The rational is to decompose the final decision into individual contributions of each given input by means of relevance backpropagation, *i.e*., trace-back contributions between layers from output to input. The result are heatmaps representing the relative relevance of each pixel to the final classification.

#### Classification Performance Metrics

To evaluate our models’ performance, accuracy, balanced accuracy, sensitivity, specificity, positive predictive value (PPV), negative predictive value (NPV), and F-score were calculated. Receiving operating characteristics (ROC) and precision-recall curves were evaluated. The area under the curve (AUC) was also assessed for both curves. Metrics were computed for the detection of living cells (in the entire dataset) and subsequent disease detection (for images with a positive classification for the presence of living cells). Confidence intervals for all the obtained metrics were also computed from the multiple model calls.

## Results

### Preliminary Analysis: Autofluorescence Intensity and Lifetime

**Figure 2:**
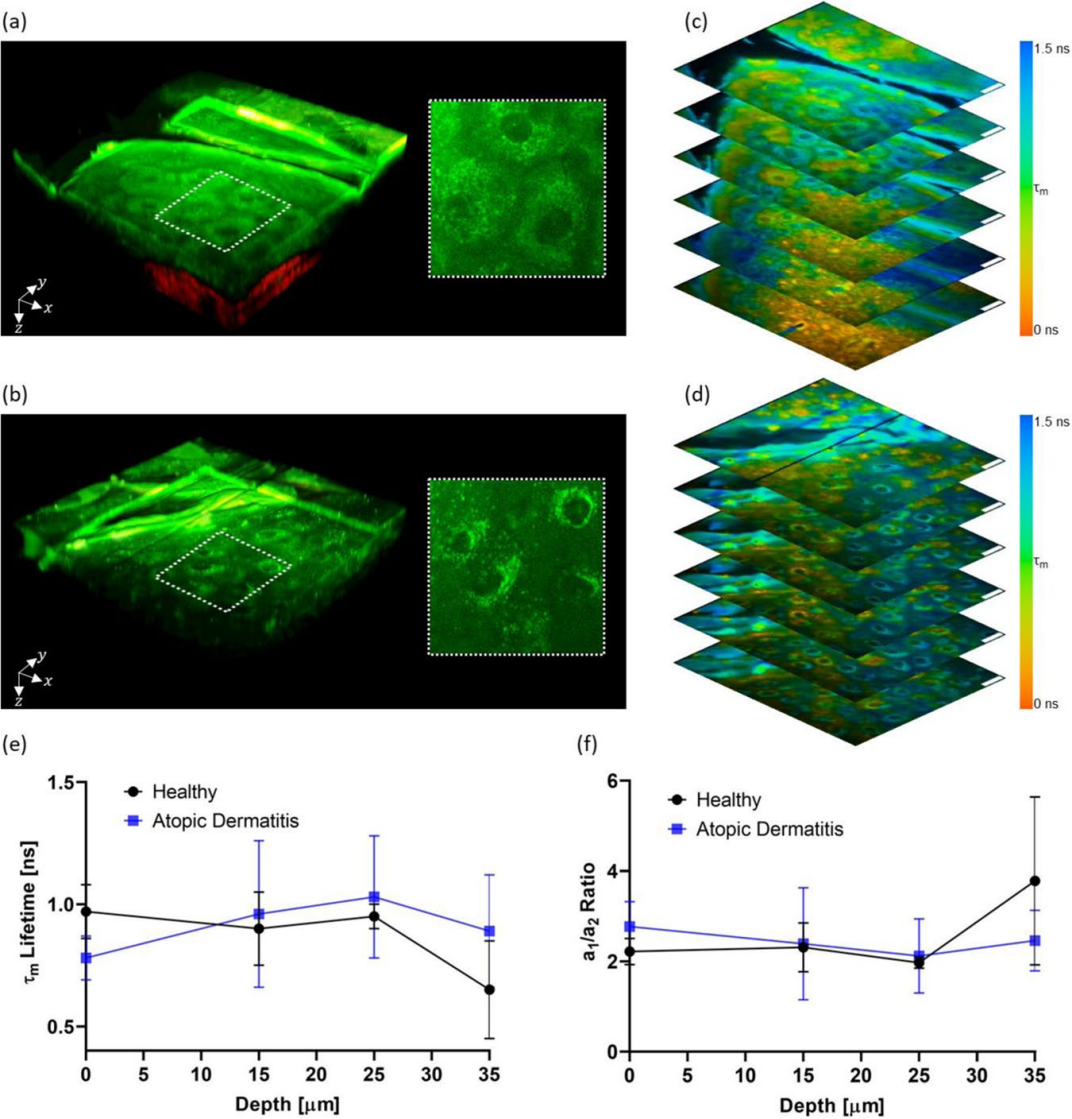
Autofluorescence intensity and lifetime analysis. Three-dimensional representation of the skin autofluorescence (green) and second-harmonic generation (red) for healthy volunteers (**a**) and atopic dermatitis patients (**b**) with highlighted regions showing cell morphology and corresponding FLIM images (c and d, respectively). FLIM images are color-coded for the mean autofluorescence (τm) lifetime as indicated in the color bar and autofluorescence intensity weighted. Scale bar = 20 µm. Changes with depth of τm lifetime (**c**) and a1/a2 ratio (**d**) in the living cell layers of healthy and atopic dermatitis diagnosed skin. FLIM images were analyzed using the commercial software SPCImage v8.0 (Becker & Hickl GmbH, Berlin, Germany).

Morphologic and metabolic AD-induced changes can be detected by MPT imaging. Figure 2a,b show tree-dimensional (3D) representations of the AF intensity (green) and SHG (red) of healthy and AD-affected skin, respectively. Highlighted regions show cell morphology. Corresponding FLIM images are shown in Fig. 2c,d. In the outermost living cell layers of the skin epidermis, AF signals are generated mainly by NAD(P)H located in the cells’ mitochondria. Therefore, changes in mitochondria arrangement within the *stratum granulosum* cells caused by AD, can be observed. In healthy skin, mitochondria appear to be evenly distributed in the cell cytoplasm (Fig. 2a). In contrast, in AD-affected skin, mitochondria appear to accumulate perinuclearly (Fig. 2b).

Figure 2e,f show the mean AF lifetimes and the ${a}_{1}/{a}_{2}$ ratio for different depths, respectively. A tendency for lower mean AF lifetimes was observed in the *stratum granulosum* (depth = 0 µm) of AD-affected skin when compared with healthy skin (Fig. 2e), however statistical significance was not reached. No changes were observed at *stratum spinosum* (depth = 15 and 25 µm). In the *stratum basale*, the detection of melanin AF leads to a decrease in the mean AF lifetime (Fig. 2e) and an increase in the ${a}_{1}/{a}_{2}$ ratio (Fig. 2f). This can be observed at approximately 35 µm depth in healthy skin. Due to the increase in epidermal thickness, in AD-affected skin, the *stratum basale* was not yet reached at this depth and such changes were not observed.

### Atopic dermatitis automatic diagnosis

From the total 3,663 patches, averaging 184 per z-stack, 1,419 were considered by a human expert to contain living cells (38.7%, averaging ~71 per z-stack), covering the *stratum granulosum*, *stratum spinosum*, and *stratum basale* layers. Images presented varying levels of quality. Nevertheless, all images were considered.

**Figure 3:**
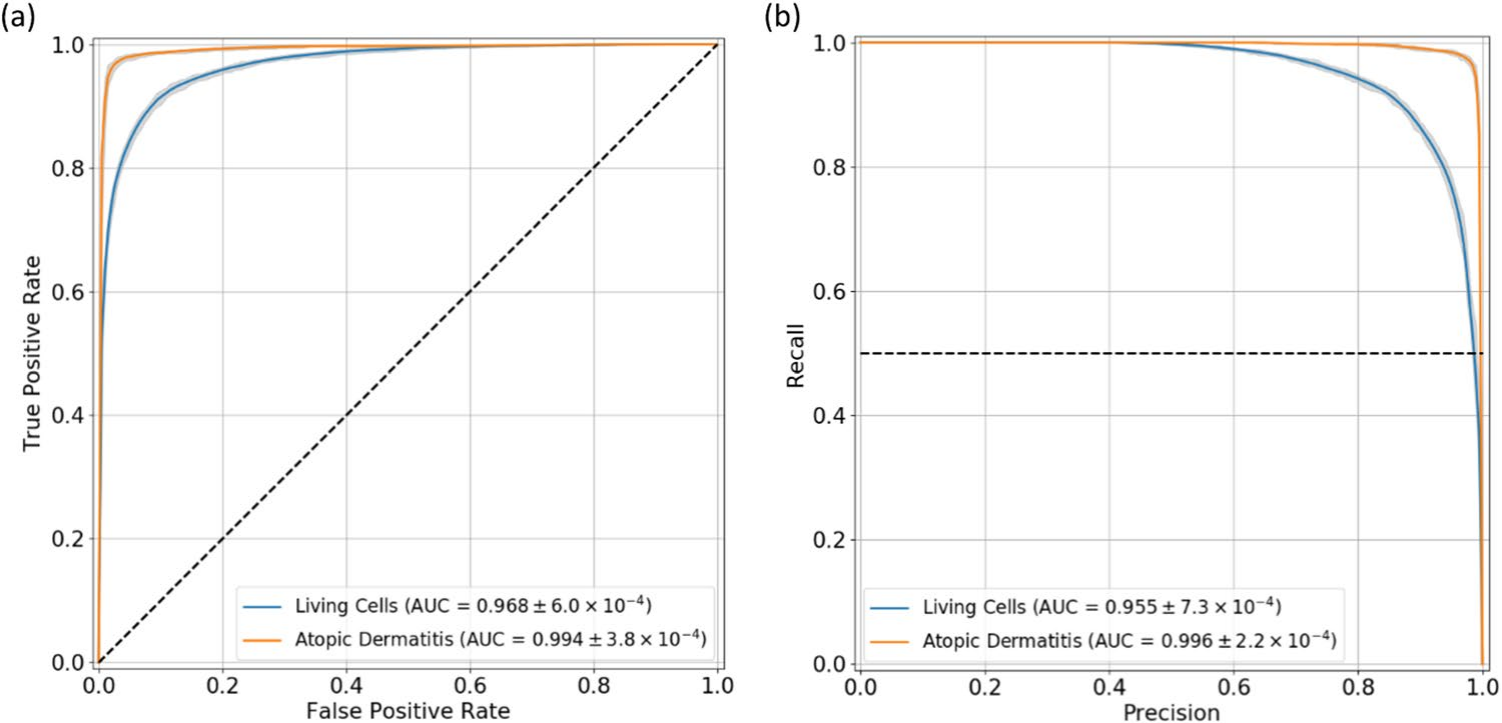
Performance curves. Receiving operating characteristics (**a**) and precision-recall (**b**) curves for the living cells and atopic dermatitis detection tasks. The range for each curve is represented in gray. The area under each curve (AUC) ± standard deviation values are showed in the graphic’s legend.

The deep learning model used to detect living cells performed well. ROC and precision-recall curves were computed and are shown in Fig. 3a,b, respectively. The AUC values achieved were 0.968 ± 6.0 × 10^−4^, and 0.955 ± 7.3 × 10^−4^, for the ROC and precision-recall curves, respectively.

In total, 1,419 images were classified as containing living cells. These were then passed to the second network (AD classification). From those, 840 of them were acquired from subjects diagnosed with AD (59.5%). The ROC and precision-recall curves for AD classification show the excellent performance achieved (Fig. 3). AUC values of 0.994 ± 3.8 × 10^−4^ and 0.996 ± 2.2 × 10^−4^ were computed, respectively.

Table 2 summarizes the remaining performance metrics for both classification tasks. Cell detection achieved an accuracy of 0.910 ± 0.002 with a higher specificity value when compared to its sensitivity (0.927 ± 0.002 vs 0.884 ± 0.004). False positives were passed down to AD classification (about 11.6% of all positives). Despite this, our approach accurately diagnosed correctly ~97% of all images, achieving a balance between sensitivity and specificity (0.966 ± 0.003 and 0.977 ± 0.003, respectively). Inter-subject and inter-stack variation were assessed by the accuracy standard deviation (Table 3). Results were consistent between z-stacks and subjects as shown by the low values obtained.

**Table 2:** Classification Performance. Performance metrics ± standard deviation values for the living cells and atopic dermatitis detection tasks. PPV – Positive predictive value; NPV – Negative predictive value.

	Living Cells	Atopic Dermatitis
Accuracy	0.910 ± 0.002	0.970 ± 0.002
Balanced Accuracy	0.906 ± 0.003	0.972 ± 0.003
Sensitivity	0.884 ± 0.004	0.966 ± 0.003
Specificity	0.927 ± 0.002	0.977 ± 0.003
PPV	0.884 ± 0.003	0.984 ± 0.002
NPV	0.927 ± 0.002	0.952 ± 0.004
F1-score	0.927 ± 0.001	0.964 ± 0.002

**Table 3:** Performance variation. Inter-stack and inter-subject accuracy standard deviation for the living cells and atopic dermatitis detection tasks.

	Inter-Stack	Inter-Subject
Living Cells	0.036	0.032
Atopic Dermatitis	0.053	0.063

### Interpretability

**Figure 4:**
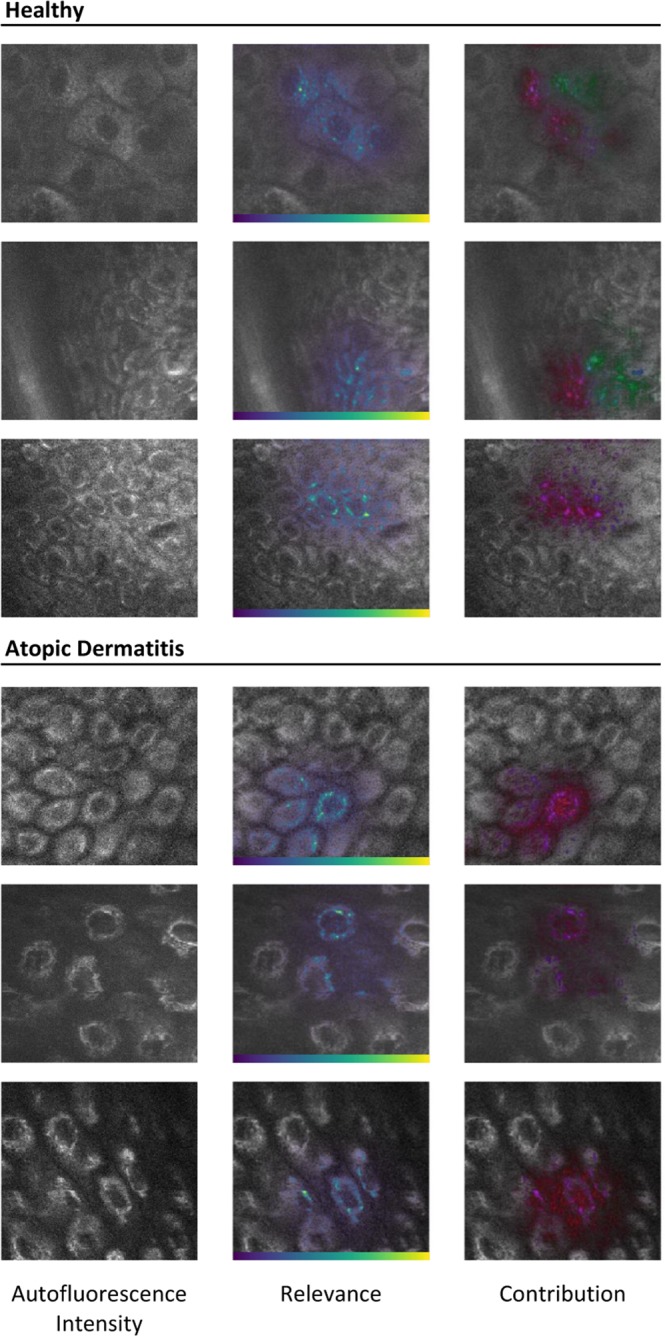
Deep Learning Interpretability. Deep Taylor decomposition relevance heatmaps for healthy and diseased subjects. Left – raw autofluorescence (AF) intensity image; Middle – AF intensity overlaid by the relative relevance heatmap color-coded according to the respective color bar; Right – AF intensity overlaid by the relative contribution of each input modality color-coded according to the RGB color model (Red-Green-Blue, as: mean AF lifetime, ratio of AF lifetime components relative contributions, and AF intensity). Transparency of overlaid heatmaps according to magnitude.

Deep Taylor decomposition was applied to AD detection. Figure 4 shows the resulting heatmaps for selected examples from healthy and AD subjects. Raw AF intensity image is shown on the left column. On the other columns, AF intensity is overlaid by semi-transparent heatmaps showing: in the middle, the relative relevance of each pixel (color-coded according to the represented color-bar), and on the right, the contribution of each input modality (RGB color model: Red - mean AF lifetime; Green - ${a}_{1}/{a}_{2}$ ratio; Blue - AF intensity).

These heatmaps show what aspects of the input make the model decide on the disease status of a given subject. As shown, cells are key for AD diagnosis. The model seems to focus on one or a few cells in each image. AF intensity and mean AF lifetime (red and blue, respectively) have a high contribution in almost all images, while the ${a}_{1}/{a}_{2}$ ratio contribution is quite dispersed. Regions with high AF intensity in the cell’s cytoplasm seem to be particularly of interest. As above mentioned, these correspond to mitochondria. The contribution of the ${a}_{1}/{a}_{2}$ ratio (displayed in green) is specifically high for some of the non-AD images and almost non-existent in AD images.

## Discussion

Using MPT, multiple layers of the skin can be investigated non-invasively with sub-cellular resolution. The nonlinear excitation of endogenous fluorophores enables its analysis at multiple depths without the need for biopsies. The morphology of each layer is assessed using the AF intensity and SHG signals from collagen fibers. In addition, due to the role NAD(P)H in the cell’s metabolic activity, the metabolism can be simultaneously evaluated. It has been previously shown that it can be used to detect inflammation in AD patients^13–16^. Nevertheless, the analysis is still time-consuming and operator dependent. Results are typically specific to a given cell layer (*stratum granulosum*), and statistical significance is not always achieved^13,15,17,35,36^.

Morphological and metabolic changes, in accordance to what has been previously reported, were observed in our preliminary analysis. Namely, mitochondria accumulate around the cell’s nucleus in the outermost cell layers of AD patients. This has been previously associated with cell inflammation^14,15^. A decrease in the mean AF lifetime and an increase in ${a}_{1}/{a}_{2}$ of s*tratum granulosum* cells, without statistical significance, was also observed, indicating decreased metabolic activity^16^. No changes were observed for the other layers.

In this study, we went a step further and implemented an end-to-end deep learning approach for the automatic classification of AD from MPT data. CNNs have been essential cogs in many of the most recent advances of computer vision and image analysis fields. Given the importance that medical imaging plays in diagnosis, deep learning has the potential to revolutionize many medical areas. In our approach, we combined AF intensity, mean AF lifetime, and ${a}_{1}/{a}_{2}$ ratio, meaning the decision algorithm used both morphological and metabolic information to model AD. The 97.0% classification accuracy achieved by our model from a small imaging area of just 62.5 × 62.5 μm^2^, shows the potential of combining state-of-the-art imaging and classification methods.

One of the concerns regarding the wide application of machine-learning in the medical field is the lack of information on why, how, and with what certainty a given classification decision is reached. This naturally hinders the possibility of experts verifying the validity of these decisions and detecting/diagnosing a mistake. Deep learning interpretability is of great importance to understand limitations and establish trust in the results.

Uncertainty estimation is of crucial importance. Variational dropout gave us a mathematical foundation to compute model uncertainty for each prediction and allowed us to establish confidence intervals. We also computed relevance heatmaps that show us what aspects of the images are important for a given classification and allow review by experts if necessary. Regions with high AF intensity located within the cytoplasm were prominent. As aforementioned, the main source of AF is NAD(P)H, a coenzyme located mainly in mitochondria. These crucial organelles in the cells’ energy production, are involved in the regulation of inflammation^37^. Therefore, the high AF intensity pattern is highly relevant for the diagnosis of AD. Metabolic information played also an important part in the model’s decision, as shown by the relative contribution heatmaps. The contribution was much more disperse when compared to AF intensity, coming from inside the cells and regions surrounding them, *i.e*., less definition. This is to be expected, since binning is required to gather enough photon counts for proper TCSPC curve fitting. The relevance maps indicate that the model is finding similar changes to those described in prior publications^13–16^, integrating and weighting them in an end-to-end manner to reach a decision on the disease status of a given subject.

In summary, we showed that MPT imaging can be combined with artificial intelligence methods to fully explore its potential. We successfully trained a CNN-based approach to detect images with living cells and discriminate AD, achieving a high sensitivity and specificity. By exploring relevance backpropagation to enhance interpretability of the proposed method, we showed that our deep learning model integrates both morphological and metabolic information provided by MPT imaging to derive a highly accurate diagnosis independent of imaging layer or region-of-interest overcoming traditional analysis methods. Moreover, the proposed approach serves not only for AD, but as a framework for the automatic diagnosis of other skin diseases using MPT imaging.
